# The Role of Polyhydroxyalkanoates in Veterinary Medicine: Biosynthesis, Material Modifications and Clinical Applications

**DOI:** 10.3390/ma19183938

**Published:** 2026-09-16

**Authors:** Adriana Elena Anita, Dragos Constantin Anita, Irina Negut, Carmen Ristoscu

**Affiliations:** 1 Regional Centre of Advanced Research for Emerging Diseases, Zoonoses, and Food Safety, “Ion Ionescu de la Brad” Iasi University of Life Sciences, 700490 Iasi, Romania; adriana.anita@iuls.ro (A.E.A.); dragos.anita@iuls.ro (D.C.A.); 2 Lasers Department, National Institute for Laser, Plasma and Radiation Physics, 409 Atomistilor Street, 077125 Magurele, Romania; negut.irina@inflpr.ro

**Keywords:** polyhydroxyalkanoates, PHBV, P4HB, biodegradable biopolymers, veterinary biomaterials, tissue engineering, drug delivery, resorbable implants, biocompatibility, sustainable biomanufacturing, wound healing

## Abstract

Polyhydroxyalkanoates (PHAs) are a structurally diverse family of microbially synthesised, biodegradable polyesters that accumulate as intracellular carbon and energy reserves under conditions of nutrient imbalance. Their combination of adjustable mechanical performance with controlled hydrolytic and enzymatic degradation, and non-toxic degradation intermediates (ex. D-3-hydroxybutyrate) has made them a longstanding candidate biomaterial for human tissue engineering, drug delivery, and resorbable implants. Comparatively, their application in veterinary medicine remains an emerging and fragmented field, despite an arguably stronger practical case: veterinary practice faces acute pressure to replace non-degradable sutures, orthopaedic hardware, and single-use plastics with materials that avoid secondary retrieval surgery, that can be produced at low cost for large-scale animal use, and that align with growing regulatory and consumer demand for sustainable animal healthcare. This review consolidates current understanding of PHA biosynthesis, covering the core: phaA-phaB-phaC pathway, medium-chain-length variants, microbial producers, feedstock flexibility, and metabolic engineering strategies for yield improvement. It also examines material modification strategies, including blending, chemical grafting, surface functionalisation, electrospinning, and additive manufacturing, used to adapt PHAs for specific veterinary form factors. The clinical and preclinical evidence base is presented in detail across wound management, orthopaedic and soft-tissue regeneration, cardiovascular tissue engineering, drug delivery, and surgical devices, with attention to species-specific considerations in companion animals, horses, and food-producing ruminants. This review relies exclusively on peer-reviewed literature for its quantitative claims, while transparently noting where veterinary-specific data are lacking, extrapolated from rodent or human models, or in need of independent verification. Persistent barriers like production cost, batch-to-batch variability, absence of veterinary-specific regulatory pathways, and limited long-term in vivo safety data in large animals are analysed critically, alongside translational opportunities including waste-feedstock valorisation, hybrid PHA/ceramic and PHA/natural-polymer composites, and stimuli-responsive formulations. We conclude that PHAs are scientifically well positioned but institutionally under-validated for veterinary translation, and we outline a concrete research agenda to close this gap.

## 1. Introduction

Polyhydroxyalkanoates (PHAs) represent a family of linear, thermoplastic polyesters synthesised by numerous bacteria, some archaea, and select recombinant eukaryotic systems as intracellular carbon- and energy-storage granules, typically induced when carbon is abundant but nitrogen, phosphorus, sulphur, or oxygen become limiting [1,2]. Structurally, PHAs are classified by the length of the pendant alkyl side chain of their monomers: short-chain-length PHAs (scl-PHAs, 3–5 carbon atoms per monomer), such as poly(3-hydroxybutyrate), PHB, and its copolymer poly(3-hydroxybutyrate-co-3-hydroxyvalerate), PHBV; and medium-chain-length PHAs (mcl-PHAs, 6–14 carbon atoms), such as poly(3-hydroxyoctanoate), PHO [3,4]. A third clinically important variant, poly(4-hydroxybutyrate), P4HB, incorporates a 4-hydroxy acid monomer and yields materials with substantially greater elasticity than PHB homopolymer [5]. This structural diversity, further expanded through copolymerisation (e.g., PHBV, poly(3-hydroxybutyrate-co-3-hydroxyhexanoate), PHBHHx), gives PHAs a broad property space spanning stiff, semicrystalline thermoplastics to soft, elastomeric films, an unusually wide design space for a single polymer family [6,7].

The biomedical rationale for PHAs rests on three main pillars: (i) biodegradability via surface hydrolysis and enzymatic (esterase/depolymerase) action. For short-chain-length PHAs such as PHB and PHBV, degradation yields D-3-hydroxybutyrate (and D-3-hydroxyvalerate in the case of PHBV), a metabolite naturally present in mammalian blood as a ketone body [8]. For poly(4-hydroxybutyrate) (P4HB), however, the degradation product is 4-hydroxybutyrate (4HB), a compound that, while endogenous (it is a precursor to the neurotransmitter GABA and a neuromodulator in the mammalian brain), follows a different metabolic and clearance pathway than ketone bodies [5,9]. This distinction is clinically relevant, as the toxicity and pharmacokinetic profiles of 4HB differ from those of D-3-hydroxybutyrate; (ii) generally sustainable biocompatibility, with limited chronic foreign-body response compared to some synthetic polyesters, although this is influenced strongly by polymer purity, crystallinity, and residual endotoxin from bacterial production [10]; and (iii) processing versatility—PHAs can be solvent-cast, melt-processed, electrospun, and 3D-printed, enabling the fabrication of films, fibres, sutures, and porous scaffolds [11].

Veterinary medicine presents a distinct and, in several respects, more compelling use case for PHA-based materials than human medicine. First, non-degradable implants and sutures in companion animals frequently necessitate second interventions for removal, which are costlier and more logistically difficult to justify in animals than in humans [12]. Second, equine practice often requires materials to be produced and deployed at large scale and low unit cost, favouring biomanufacturing routes that can exploit agricultural or agro-industrial waste streams as PHA feedstocks, an area of active and ongoing research interest independent of veterinary applications [13,14]. Third, rising concern over antimicrobial resistance in food-animal production has intensified interest in biomaterial-based, non-antibiotic strategies for local infection control (e.g., antimicrobial-loaded coating), for which PHA carriers are mechanistically well-suited given their adjustable, sustained-release degradation kinetics [15]. Fourth, environmental sustainability considerations, including the volume of single-use plastic and non-degradable medical waste generated in agricultural and companion-animal care, add a life-cycle argument for biodegradable veterinary materials that is largely absent from the human-medicine literature [16].

Despite this rationale, the veterinary PHA literature remains considerably thinner and more fragmented than its human-biomedical counterpart. A substantial share of what is described in preliminary or non-peer-reviewed summaries as “veterinary PHA studies” in fact extrapolates from rodent, ovine, or bovine work conducted primarily as a proxy for human clinical translation (e.g., sheep and lamb models used to validate tissue-engineered heart valve or vascular scaffolds intended for paediatric human patients) rather than as veterinary-clinical research per se [17,18]. This review is explicit about that distinction throughout: findings from large-animal models used as a human-translational surrogate are presented as such and are distinguished from studies whose primary endpoint was veterinary clinical benefit.

This review synthesises current knowledge across three domains: (1) the biosynthetic and metabolic-engineering basis for PHA production, with emphasis on the factors governing yield, monomer composition, and material-relevant properties; (2) the material modification strategies: physical, chemical, and processing-based used to adapt PHAs for specific veterinary form factors and species; and (3) the clinical and preclinical evidence for PHA use across wound care, orthopaedic and soft-tissue regeneration, cardiovascular applications, drug delivery, and surgical devices in companion animals, horses, and ruminants. We conclude by critically evaluating key translational barriers and a prioritised research agenda.

The review proposes an integrated materials-to-clinical-translation perspective in which PHA chemistry and material architecture determine behaviour and mechanical persistence, while degradation kinetics interacts with host responses and tissue remodeling to determine biological performance and functional lifetime. This perspective highlights the substantial potential of PHAs for veterinary medicine while also demonstrating that their transition from promising biomaterials to clinically established technologies will depend on standardised characterisation, target-species validation, long-term safety studies, cost-effective manufacturing, and rigorously designed veterinary clinical trials.

## 2. Materials and Methods

The literature review conducted as part of this study involved a comprehensive investigation of polyhydroxyalkanoates, with particular focus on their biosynthesis, physicochemical and biological properties, material modifications, biodegradability, biocompatibility, and applications in veterinary medicine. The literature search was conducted using the PubMed, Scopus and Web of Science databases. The following keywords and their combinations were used: “polyhydroxyalkanoates”, “PHA”, “polyhydroxybutyrate”, “PHB”, “biosynthesis”, “microbial production”, “biopolymers”, “biodegradable materials”, “biocompatibility”, “material modification”, “drug delivery”, “tissue engineering”, “wound healing”, “orthopaedics”, “veterinary medicine”, “veterinary applications”, “animal health”, “implants”, and “biomedical applications”. Relevant combinations of these terms using Boolean operators were applied to identify publications addressing the development and application of PHA-based materials in veterinary and related biomedical fields. Only research and review papers published in English were included. This work will principally focus on the most relevant research published between 1984 and 2026.

## 3. PHAs in the Context of Existing Biodegradable Polymers for Veterinary Use

Before assessing the unique value proposition of PHAs in veterinary medicine, it is important to position them relative to the biodegradable polymers already in clinical veterinary use. Several absorbable synthetic polymers have long-established track records in veterinary surgery, and their properties, costs, and limitations provide an important benchmark against which PHA-based devices must be compared.

Polydioxanone (PDS) is a synthetic absorbable monofilament suture widely used in veterinary practice. PDS retains tensile strength for approximately 6–8 weeks post-implantation and undergoes complete absorption over approximately 180 days, the longest absorption timeline among synthetic absorbable sutures used in routine veterinary surgery [4,5]. PDS is valued for its monofilament construction (reduced tissue drag and bacterial adherence), excellent handling characteristics, and predictable degradation profile [4]. However, PDS is derived from petrochemical precursors, is not produced from renewable resources, and its degradation products are not naturally occurring metabolites. PHAs, in contrast, degrade to D-3-hydroxybutyrate—a naturally occurring ketone body—and can be produced from renewable feedstocks.

Polyglycolic acid (PGA) and poly (lactic-co-glycolic acid) (PLGA) are additional workhorse biodegradable polymers in veterinary and human medicine. PGA sutures absorb within 60–90 days via hydrolysis [6] and are used in tissues where rapid healing is expected [6]. PLGA, widely used for drug delivery and tissue engineering scaffolds, offers adapted degradation kinetics through variation of the lactic-to-glycolic acid ratio. Both polymers have well-characterised safety profiles and regulatory acceptance. However, PGA and PLGA degrade via bulk hydrolysis, producing acidic degradation products (glycolic and lactic acid) that can create a local acidic microenvironment, potentially contributing to inflammatory responses or autocatalytic degradation, phenomena less commonly reported for surface-eroding PHAs.

Polylactic acid (PLA) and its stereoisomers (PLLA, PDLA) are used in veterinary orthopaedics, including resorbable implants for tibial tuberosity advancement (TTA) in canine cruciate ligament repair [7]. PLA implants for TTA provide good functional results, presenting an acceptable rate of complications, faster bone healing of the osteotomy gap, and clinical recovery times similar to metallic implants [7]. PLA has been evaluated in horses as well, with studies demonstrating prolonged biocompatibility and biodegradation [8]. PLA is produced from renewable resources (corn starch, sugarcane) and has a well-established regulatory and manufacturing infrastructure. However, PLA is less flexible than P4HB, degrades more slowly in some formulations (complete absorption in up to 4 years in canine models [10]), and its degradation products are not naturally occurring metabolites to the same extent as PHAs.

Polycaprolactone (PCL) is a slowly degrading polyester used in some veterinary drug-delivery and tissue-engineering applications. PCL has been investigated for scaffolds for tissue engineering, wound dressings, nerve regeneration devices, and drug-delivery systems [11]. Its degradation half-life is measured in years, limiting its use to applications requiring very long-term stability [11].

Against this backdrop, PHAs offer several potential advantages: degradation to naturally occurring metabolites; a wider range of mechanical properties ranging from stiff thermoplastics (PHB) to elastomers (P4HB, PHBHHx); and the ability to be produced from a diverse array of waste feedstocks. However, these advantages must be weighed against PHA’s higher production cost, less extensive regulatory precedent in veterinary medicine, and the need for dedicated veterinary clinical validation. Importantly, PHA degradation is not uniform across polymer classes and is strongly dependent on copolymer composition, crystallinity, molecular weight, morphology, implant geometry, and the local implantation environment. The choice between PHA and existing biodegradable polymers will ultimately depend on the specific application: P4HB’s exceptional elasticity and strength retention make it uniquely suited to sutures and soft-tissue meshes, where it competes directly with PDS; the relatively high crystallinity of PHB generally contributes to slower degradation, whereas incorporation of 3-hydroxyvalerate units in PHBV can reduce crystallinity and modify chain mobility and degradation kinetics. Medium-chain-length PHAs generally exhibit greater chain flexibility and lower crystallinity, resulting in degradation and mechanical-retention profiles that differ substantially from those of PHB and PHBV. PHB/HA composites offer an osteoconductive, resorbable alternative to PLA in orthopaedics; and PHA microspheres provide a drug-delivery platform that may offer advantages over PLGA in terms of degradation product profile. In addition, implant dimensions, porosity, surface-area-to-volume ratio, and local tissue conditions, including enzymatic activity, inflammatory responses, pH, fluid exchange, and cellular interactions, can further modulate degradation in vivo. These comparative considerations should inform the selection of PHA formulations for veterinary device development, yet they are rarely articulated in the existing literature. A comparative overview of biodegradable polymers currently used or under investigation in veterinary medicine is provided in Table 1, highlighting the respective advantages, limitations, and clinical applications of each material relative to PHAs.

## 4. Biosynthesis of PHAs

### 4.1. Microbial Producers and the Core Biosynthetic Pathway

PHAs are synthesised by a taxonomically broad range of Gram-negative and Gram-positive bacteria, with *Cupriavidus necator* (formerly *Ralstonia eutropha*, *Alcaligenes eutrophus*) remaining the most extensively characterised and industrially exploited producer of scl-PHAs, alongside recombinant *Escherichia coli*, *Bacillus* spp., *Halomonas* spp. (notably relevant for low-cost, non-sterile, high-salinity fermentation), and various *Pseudomonas* spp., which are the principal natural producers of mcl-PHAs [1,15,16].

**Figure 1:**
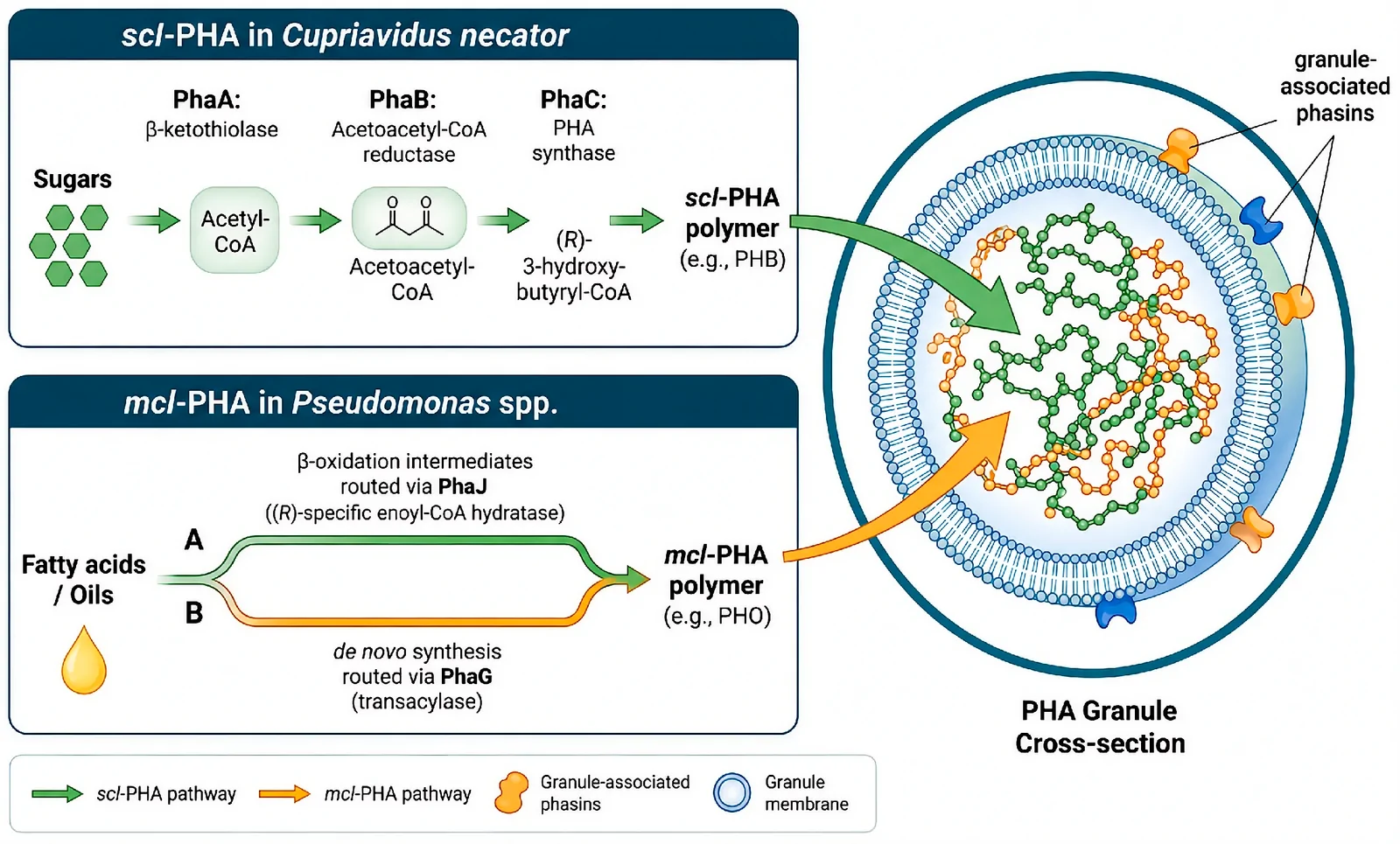
Schematic of the core PHA biosynthetic pathway in *Cupriavidus necator* and *Pseudomonas* spp., showing PhaA/PhaB/PhaC (scl-PHA) and PhaJ/PhaG (mcl-PHA) enzymatic routes from acetyl-CoA/fatty acid precursors to granule formation. The image was created by using FigureLabs (cloud-based platform, FigureLabs, available online: https://figurelabs.ai/).

The standard scl-PHA pathway in *Cupriavidus necator* proceeds through three enzymatic steps: (i) β-ketothiolase (PhaA) condenses two molecules of acetyl-CoA into acetoacetyl-CoA; (ii) NADPH-dependent acetoacetyl-CoA reductase (PhaB) reduces this intermediate to (R)-3-hydroxybutyryl-CoA; and (iii) PHA synthase (PhaC) polymerises the monomer into PHB, which accumulates as cytoplasmic granules bound by a phospholipid monolayer and granule-associated proteins (phasins) [2,17]. For mcl-PHAs, monomers are instead derived from fatty acid β-oxidation or de novo fatty acid synthesis intermediates, channelled into the PHA pathway via (R)-specific enoyl-CoA hydratase (PhaJ) or the transacylase PhaG, respectively, before polymerisation by a distinct class of PHA synthases with broader substrate specificity [18,20]. A schematic representation of the core PHA biosynthetic pathway in *C. necator* and *Pseudomonas* spp. is shown in Figure 1. The monomer composition dictates the resulting polymer’s crystallinity, glass transition temperature, and mechanical behaviour; copolymers such as PHBV are produced by supplying propionate or related odd-chain precursors alongside glucose, incorporating 3-hydroxyvalerate units that disrupt PHB crystallinity and confer greater flexibility and lower melting temperature [21,22].

### 4.2. Factors Governing Yield and Material Properties

PHA yield and monomer composition are influenced by the carbon source, nutrient stoichiometry, oxygen availability, and fermentation strategy. Nitrogen or phosphorus limitation under carbon excess is the classical trigger for PHA accumulation; fed-batch fermentation strategies that decouple biomass growth from the polymer-accumulation phase remain the industrial standard for achieving high cell-dry-weight PHA content (frequently reported in the 60–80% CDW range for optimised *C. necator* and recombinant *E. coli* systems) [23,24]. Molecular weight, which strongly influences mechanical strength and degradation rate, is affected by fermentation duration, PhaC kinetics, and the presence of chain-transfer agents; higher-molecular-weight PHAs generally show greater tensile strength but slower, more surface-limited degradation [25].

Metabolic engineering has targeted several bottlenecks: overexpression of phaC and phaAB to increase flux into the pathway; deletion or downregulation of intracellular PHA depolymerases (phaZ) to prevent premature mobilisation of stored polymer; and heterologous expression of PHA operons in fast-growing, well-characterised chassis strains such as *E. coli* to simplify downstream purification (since *E. coli* lacks native depolymerase activity and produces PHA with fewer co-extracted endotoxin-adjacent contaminants than some native producers, though endotoxin removal remains an essential purification step for any biomedical-grade PHA) [18,26]. CRISPR-based genome editing and synthetic promoter systems have more recently been applied to fine-tune expression stoichiometry between PhaA, PhaB, and PhaC, addressing the metabolic burden and flux imbalances that limit yield in wild-type strains [27].

Recent metabolic engineering efforts have yielded significant improvements in PHA production efficiency. A comprehensive review by Hectors et al. [11] detailed advanced strategies in *Cupriavidus necator*, including optimisation of central carbon flux, redox and cofactor balancing, adaptation to oxygen-limiting conditions, and fine-tuning of granule-associated protein expression. Notably, enzyme engineering and the establishment of novel artificial pathways have enabled biosynthesis from unrelated single-carbon sources. In a parallel development, Gao et al. [28] constructed the first genome-scale metabolic model for *Haloferax mediterranei* (iHM951), comprising 1862 reactions and 1827 metabolites. Overexpression of triosephosphate isomerase (TpiA) in this archaeon led to a 26% increase in biomass and a 47% enhancement in PHBV production [28]. These advances position *C. necator* and *H. mediterranei* as increasingly valuable microbial chassis for industrial-scale biopolymer production [28].

### 4.3. Feedstock Sustainability and Cost

A major driver of current PHA research, directly relevant to veterinary translation given the cost sensitivity of livestock applications, is the substitution of refined carbon sources (glucose) with lower-cost, waste-derived feedstocks: crude glycerol from biodiesel production, whey permeate, waste cooking oils and fatty acid streams (favouring mcl-PHA producers such as *Pseudomonas* spp.), lignocellulosic hydrolysates, and municipal or agro-industrial wastewater processed via mixed microbial consortia [29,30,31]. Mixed-culture PHA production, in which unsterile, feast–famine-fed activated-sludge-type consortia are used instead of pure axenic cultures, is an active area of process research aimed at reducing sterilisation and substrate costs, though it introduces greater batch-to-batch compositional variability, a concern that is particularly acute for any biomedical-grade application, where consistency in molecular weight, monomer ratio, and endotoxin content is a regulatory prerequisite [32]. PHA production costs remain higher than those of commodity plastics and are also generally higher than poly(lactic acid) (PLA) on a per-kilogram basis at current production scales, although the gap has narrowed with process intensification and feedstock innovation; closing it further is widely regarded as the single most consequential lever for veterinary-scale (particularly livestock-scale) adoption [33,34].

A review by Pan et al. [35] systematically summarised three principal cost-reduction strategies: (i) utilising waste resources as feedstocks, (ii) employing extremophiles as producer strains to enable non-sterile production, and (iii) applying genetic engineering to enhance PHA yields. The economic analysis of PHA production from various waste streams was systematically quantified [35]. In a complementary development, Sankhyan et al. [36] demonstrated that waste cooking oil (WCO) serves as an excellent, cost-effective carbon source for PHA production, with WCOs shown to be particularly suitable for medium-chain-length PHA production by *Pseudomonas* species [36]. Additionally, Jaffur et al. [37] explored innovative approaches to enhance PHA production through the utilisation of organic waste substrates, including chitosan, lignin, and cellulose derivatives, which can be incorporated as additives or blending agents to improve functional properties [37].

### 4.4. Structure–Property Relationships Relevant to Veterinary Materials

PHB homopolymer is a highly crystalline (55–70% crystallinity), relatively stiff and brittle thermoplastic with a melting temperature around 170–180 °C and a glass transition temperature near 0–5 °C, properties that make it prone to post-processing embrittlement via secondary crystallisation but well suited, in principle, to load-bearing or rigid applications [38,39]. Incorporation of 3-hydroxyvalerate (PHBV) or 4-hydroxybutyrate (P4HB) reduces crystallinity and increases elongation at break and impact toughness, at the cost of reduced tensile modulus [14,40]. Throughout this review, PHBV refers to copolymers containing 10–20% 3-hydroxyvalerate (HV), unless otherwise specified.

It is important to note that the mechanical properties of P4HB are highly processing-dependent. In bulk, unoriented form (e.g., solvent-cast film), P4HB exhibits elastomeric behaviour with tensile strength of approximately 30–40 MPa and elongation of 100–300%, consistent with its reduced crystallinity and modulus [14]. However, when processed into oriented, fibre-drawn forms (e.g., commercial sutures such as MonoMax^®^, B. Braun Surgical, S.A., Barcelona, Spain), the polymer chains align along the fibre axis, leading to a dramatic increase in tensile strength to 500–800+ MPa and a reduction in elongation at break to approximately 15–30% [5,41]. This distinction, between bulk elastomeric properties and device-specific oriented fibres, is very important when interpreting the data in Table 2. While bulk P4HB has a tensile modulus of approximately 0.05–0.1 GPa—substantially lower than PHB (3.0–3.5 GPa)—orientation processing can transform it into a high-strength fibre suitable for load-bearing sutures and meshes. Care must therefore be taken when comparing values across different material states and processing conditions [14].

PHBHHx, produced by strains such as *Aeromonas hydrophila* or engineered *C. necator*, similarly yields a more elastomeric material (tensile modulus: 0.2–0.5 GPa) with improved processability and reduced brittleness relative to PHB [42]. These structure–property relationships underpin the rational selection of PHA grade for a given veterinary application: high-crystallinity PHB or PHB/ceramic composites are the logical starting point for orthopaedic and load-bearing applications, whereas P4HB, PHBV, or PHBHHx are better suited to sutures, soft-tissue meshes, wound dressings, and elastomeric scaffolds [41,43].

Degradation behaviour, governed by crystallinity, molecular weight, surface area, and the local enzymatic/microbial environment, is the second key design parameter. In vivo, PHA degradation may involve surface-associated processes, with the relative contribution of hydrolytic, enzymatically mediated, and cell-associated mechanisms depending on the specific PHA composition, formulation, device architecture, and implantation site. Interactions with macrophages and other tissue-associated cells, as well as exposure to tissue-derived enzymes, may contribute to polymer chain cleavage in certain biological contexts; however, these mechanisms should not be considered universal across all PHA systems.

Thus, implant lifetime cannot be reliably predicted from environmental biodegradation data alone and must be established through dedicated in vivo degradation studies for each formulation and implantation site [44,45].

A necessary translational consideration often overlooked in the biomaterial’s literature is that in vivo degradation kinetics do not scale linearly with body mass. The rate of hydrolytic and enzymatic erosion of a PHA implant depends on local tissue perfusion, immune cell density, and metabolic activity—all of which follow allometric scaling rules across mammalian species. For example, basal metabolic rate scales as body mass^0.75^ (Kleiber’s law), and tissue turnover rates are generally faster in smaller animals. A PHA scaffold that resorbs over 6 months in a murine model may take significantly longer in a horse, and conversely, an implant designed for human use may degrade more rapidly in a dog with a higher mass-specific metabolic rate. Therefore, the half-life ranges presented in Table 2 are species- and site-specific approximations, derived from the sparce available in vivo data (predominantly rodent and, in some cases, ovine models). They should not be treated as universal material constants. Quantitative translation of degradation timelines to veterinary species requires allometric scaling—adjusting time estimates by metabolic mass (kg^0.75^) or by species-specific tissue turnover rates—or, ideally, direct empirical validation in the target species [45,46].

### 4.5. Stereochemical Purity and Its Implications for Degradation

An important but often overlooked factor involved in the in vivo degradation behaviour of PHAs is their stereochemical configuration. Naturally occurring bacterial PHAs are isotactic and enantiomerically pure, consisting exclusively of (R)-3-hydroxyalkanoate repeating units. This stereoregularity is a direct consequence of the substrate specificity of the PHA synthase enzyme, which polymerises only (R)-configured monomers.

However, PHAs can also be produced by chemical ring-opening polymerisation (ROP) of racemic or enantiopure lactones. Depending on the catalyst and reaction conditions, ROP can yield polymers with isotactic, syndiotactic, or atactic stereochemical configurations. While chemosynthesis offers advantages in monomer diversity and scalability, it introduces a fundamental translational risk: the stereospecificity of the enzymes that degrade PHAs in vivo.

Mammalian esterases and bacterial PHA depolymerases are highly stereospecific—they recognise and hydrolyse only (R)-configured hydroxyalkanoate units. (S)-configured units, or racemic sequences, are poor substrates for these enzymes. Consequently, a chemosynthetic PHA containing (S) or racemic segments will exhibit slower, less complete, and less predictable degradation than its enantiopure bacterial counterpart. In extreme cases, atactic P(3HB) has been shown to display essentially no weight loss after 21 days of enzymatic exposure, whereas isotactic bacterial PHB degrades rapidly under identical conditions.

This stereochemical dependence has direct clinical and regulatory implications for veterinary PHA devices:

• Resorption timelines derived from bacterial PHA studies cannot be assumed to apply to chemosynthetic PHA formulations unless the stereochemistry is explicitly characterised and shown to be equivalent.
• Impurity profiles—the presence of even small amounts of (S)-units can create “dead zones” that resist enzymatic attack, leading to non-uniform surface erosion and potentially delayed or incomplete implant resorption.
• Regulatory submissions for PHA-based devices must therefore include stereochemical characterisation (e.g., by chiral chromatography or NMR) as part of the material specification, and degradation studies must be performed on the final formulated device, not on a generic bacterial PHA standard.

For the purposes of this review, all cited in vivo degradation data and commercial device approvals (e.g., P4HB sutures and meshes) pertain to bacterially derived, enantiopure (R)-PHAs. The discussion of degradation kinetics in Table 2 and throughout the manuscript should be understood in this context. Any future development of chemosynthetic PHA variants for veterinary use must include dedicated stereochemical and degradation validation before claims of equivalence to bacterial PHA can be made.

## 5. Material Modifications of PHAs for Veterinary Applications

Native PHAs rarely meet the full property profile demanded by a specific veterinary device, and a substantial modification toolkit has been developed to adapt them.

### 5.1. Physical Blending and Composite Formation

Blending PHAs with other biodegradable or bioactive polymers is the most widely used strategy to offset native limitations such as brittleness (PHB) or excessive hydrophobicity. Blends with chitosan, collagen, gelatine, or poly(lactic-co-glycolic acid) (PLGA) improve hydrophilicity, cell adhesion, and processability, while introducing challenges related to phase compatibility and potential mechanical heterogeneity at the blend interface [47]. Composite formation with hydroxyapatite (HA) or other calcium phosphate ceramics is a particularly relevant strategy for orthopaedic veterinary applications, since HA incorporation increases stiffness and osteoconductivity, bringing the composite’s mechanical profile closer to that of cancellous or cortical bone while retaining the resorbability of the polymer matrix [48].

A comprehensive 2025 review by Cichoń and Guzik [49] examined the current advancements in bioceramic/polyhydroxyalkanoate (BioC/PHA) composites, emphasising their growing role in biomedical applications. The integration of PHAs with bioceramics like hydroxyapatite or bioglass offers a unique synergy, combining the structural integrity of ceramics with the tuneable properties of PHAs [49]. Such composites demonstrate significant promise in bone tissue engineering, cartilage repair, and drug-delivery systems, where they support cell attachment, proliferation, and targeted therapeutic release. The review also highlighted advances in functionalisation, such as drug incorporation and bioactive coatings, as pathways to customised therapeutic solutions.

### 5.2. Chemical Functionalisation

Surface and bulk chemical modification—via plasma treatment, aminolysis, or covalent grafting—is used to introduce reactive functional groups (carboxyl, amine, hydroxyl) onto otherwise chemically inert PHA surfaces, enabling subsequent conjugation of cell-adhesion peptides (e.g., RGD sequences), growth factors, or antimicrobial agents [50,51]. Surface hydrophilisation (via plasma treatment, UV/ozone exposure, or PEGylation) improves protein adsorption and cell attachment on otherwise poorly wettable PHA surfaces, an important consideration since native PHB and PHBV surfaces are relatively hydrophobic and can show suboptimal initial cell attachment compared to some synthetic polyesters [52].

### 5.3. Processing Techniques for Veterinary Form Factors

**Figure 2:**
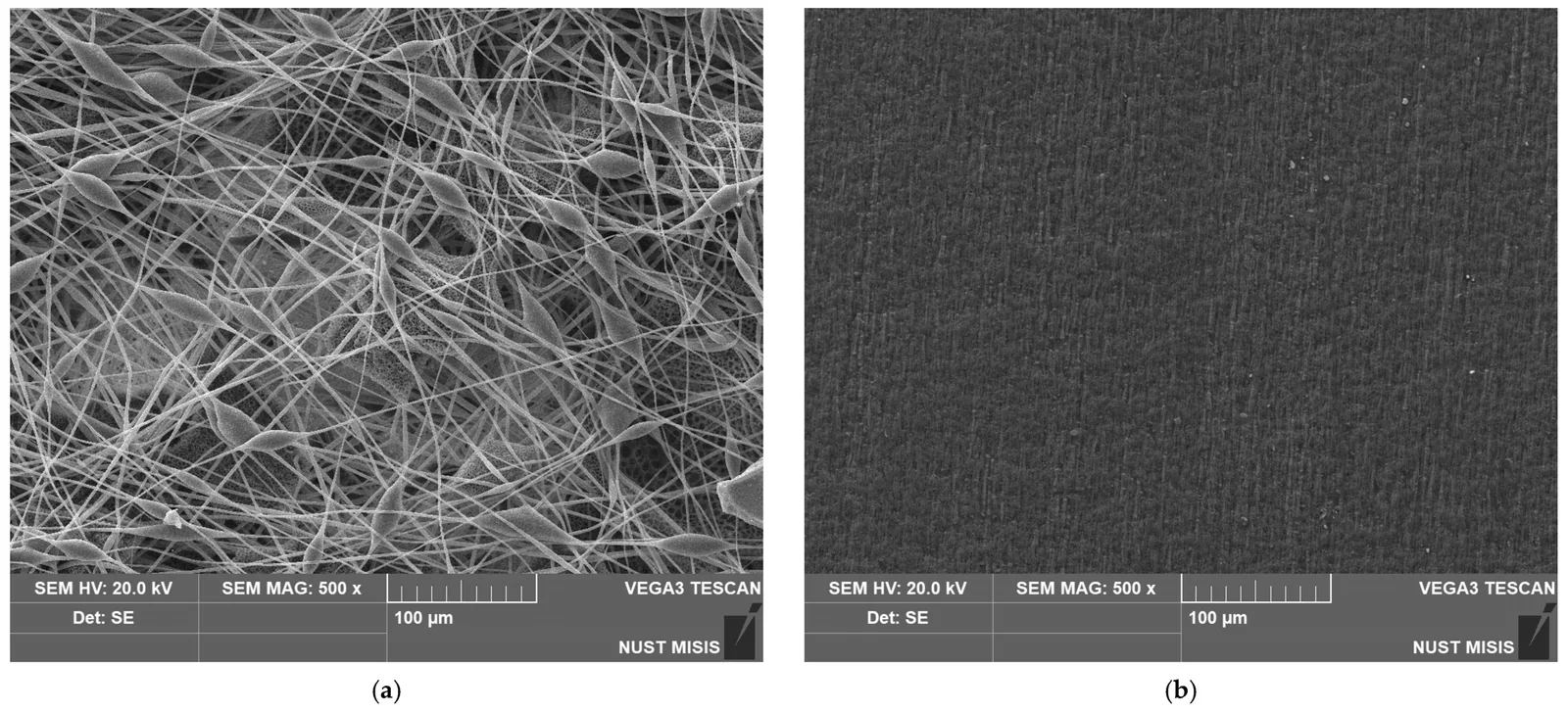
SEM images of the materials based on PHB obtained by different methods: (**a**) electrospinning, (**b**) pressing. Reprinted from Gasparyan et al. [55], licensed under CC BY 4.0.

• Electrospinning produces nanofibrous, extracellular-matrix-mimicking scaffolds with high surface-area-to-volume ratio, well suited to wound dressings and soft-tissue scaffolds; fibre diameter and porosity are controlled through solution viscosity, applied voltage, and collector geometry [9,53,54,55].
• Solvent casting/particulate leaching and freeze-drying generate porous, sponge-like scaffolds for tissue-engineering applications requiring cell infiltration [54].
• Compression pressing enables the fabrication of uniform, dense and thin PHB films using a laboratory hydraulic press equipped with heated plates. Controlled temperature and pressure allow reproducible film dimensions and morphology, providing compact structures suitable for subsequent functionalisation or biomedical testing [55].
• Melt extrusion and subsequent fibre processing is used for monofilament sutures and films, exploiting PHA thermoplasticity, though thermal degradation and secondary crystallisation during processing must be carefully controlled given PHB’s relatively narrow processing window between melting and thermal decomposition temperatures [38,39].
• Additive manufacturing (fused deposition modelling and related 3D-printing techniques) is an increasingly explored route for patient- (or animal-) specific, geometrically complex scaffolds, including composite PHA/ceramic filaments for orthopaedic applications; this remains a comparatively young area for PHAs relative to PLA, in part because of PHA’s narrower thermal processing window and lower melt strength [53]. The morphological diversity achievable through these processing techniques is illustrated in Figure 2.

### 5.4. Optimising Degradation and Bioactivity to the Clinical Context

Fast-remodelling soft tissues (skin, subcutaneous tissue) generally favour lower-crystallinity, more hydrophilic formulations with degradation in the order of weeks; load-bearing bone applications require materials whose mechanical integrity is retained over the months typically needed for osseous union, which in turn constrains the degree of hydrophilic modification and porosity that can be tolerated without compromising early mechanical strength [43,56,57]. Stimuli-responsive PHA-based systems, incorporating thermos-responsive or pH-responsive comonomers or crosslinkers, have been explored primarily at proof-of-concept level for triggered drug release, and represent a promising but still early-stage direction rather than an established veterinary technology [58].

### 5.5. Comparative Strengths and Limitations of Modification Strategies

**Figure 3:**
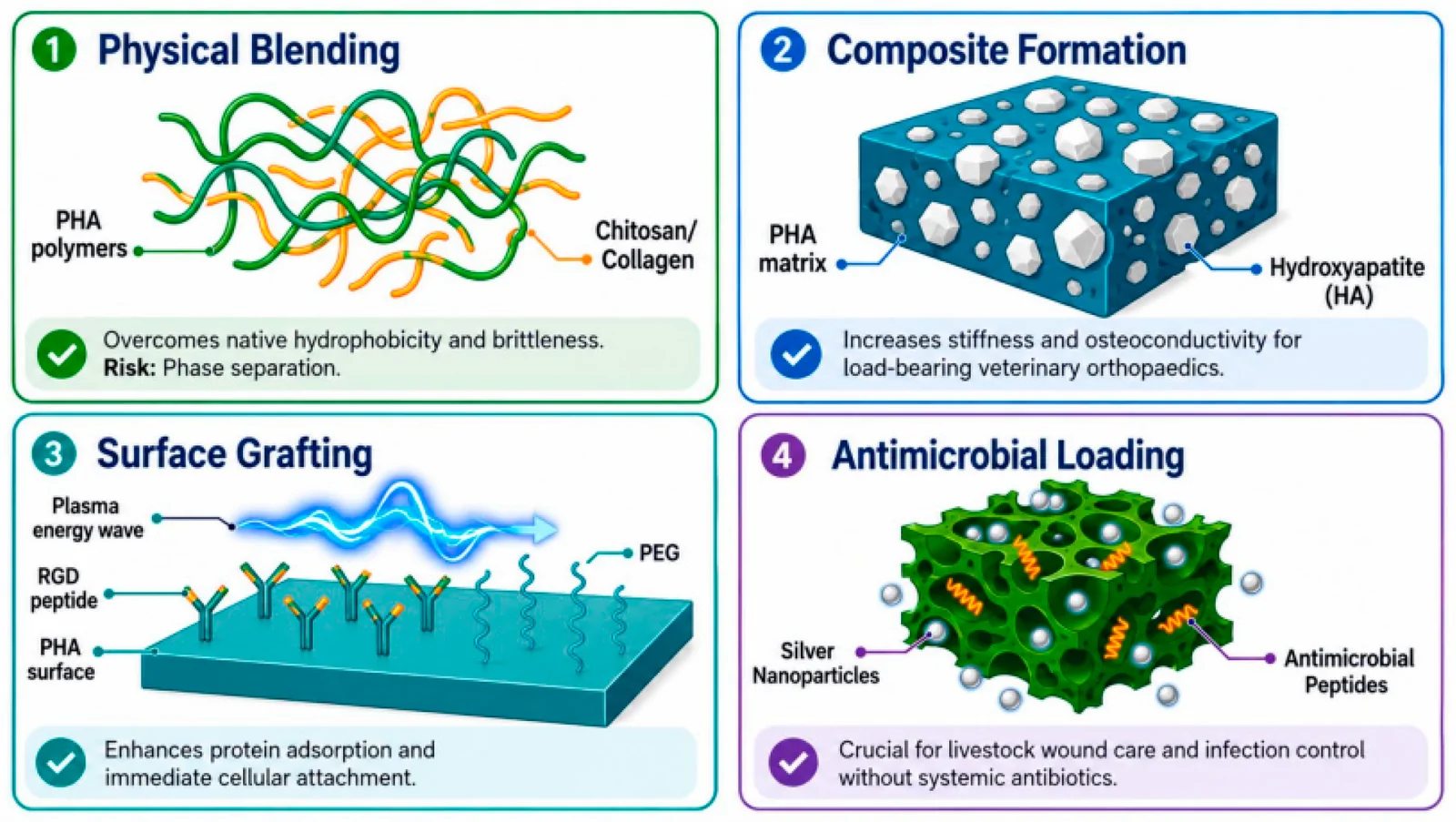
Overview of physical and chemical modification strategies applied to PHAs (blending, HA/ceramic compositing, surface grafting with RGD peptides or PEG, antimicrobial nanoparticle loading). The image was created by using FigureLabs (cloud-based platform, FigureLabs, available online: https://figurelabs.ai/).

Physical blending is a simple and scalable strategy; however, thermodynamic incompatibility between mixed components can result in phase separation, leading to heterogeneous microstructures and compromised mechanical performance. Chemical grafting and crosslinking offer precise, durable functionalisation but add processing complexity, cost, and the risk of introducing cytotoxic residues (unreacted crosslinkers, solvents) if reaction and purification conditions are not tightly controlled—an important quality-control consideration for any material intended for implantation [47,50]. No single strategy is universally superior; successful veterinary PHA devices to date generally combine two or more approaches (e.g., a PHBV/HA composite that is also surface-functionalised for cell adhesion) tailored to the specific target application. A visual overview of these modification strategies is provided in Figure 3. A summary of PHA-modification strategies, their primary mechanisms, targeted property changes, and principal limitations is presented in Table 3.

### 5.6. Sterilisation Considerations for PHA-Based Veterinary Devices

Sterilisation is an important and often underappreciated step in the translation of any biodegradable polymer from laboratory-scale material to clinically implantable device. For PHA-based veterinary devices, the choice of sterilisation method must balance effective microbial inactivation against preservation of polymer molecular weight, mechanical integrity, and degradation kinetics, properties that are essential for device function and are directly influenced by sterilisation conditions.

The most commonly investigated sterilisation methods for PHAs are ethylene oxide (EtO) gas and gamma irradiation. Marois et al. systematically evaluated the effect of both methods on poly(3-hydroxyoctanoate) (PHO), a medium-chain-length PHA, demonstrating that EtO sterilisation at 38 °C preserved the chemical and physical characteristics of PHO films, with no residual ethylene oxide detected by head-space chromatography [57]. In contrast, gamma radiation at 2.5 Mrad caused random chain scission and physical cross-linking, resulting in significant modifications to both structural and tensile properties, including decreases in weight-average and number-average molecular weight and melting temperature, alongside increases in heat of fusion and tensile strength [58]. Such effects are consistent with the ability of high-energy ionising radiation to generate free radicals within aliphatic polyesters, resulting in competing chain-scission and cross-linking reactions. These findings are consistent with the broader polymer literature, where high-energy irradiation is known to induce free-radical-mediated degradation in aliphatic polyesters.

For PHB and PHBV homopolymers and copolymers, ionising radiation can induce chain scission and modification of molecular weight, with subsequent consequences for crystallinity, thermal behaviour, mechanical performance, and radiation-induced decreases in tensile strength. Changes in crystallinity may also occur as a secondary consequence of chain scission, because shorter chains can reorganise and recrystallise during or after irradiation. Electron beam (e-beam) sterilisation has emerged as a potential alternative, though the effects of e-beam on PHA properties are less extensively characterised than those of gamma irradiation [59].

Low-temperature plasma sterilisation, including hydrogen-peroxide-based plasma systems, offers an attractive option for temperature-sensitive polymeric devices because sterilisation is performed at relatively low temperatures and without the prolonged thermal and aqueous exposure associated with steam.

From a veterinary translational perspective, these distinctions are consequential. Gamma sterilisation, while rapid and penetrating, may be unsuitable for PHA devices whose mechanical performance depends essentially on high molecular weight, such as orthopaedic fixation pins, sutures, and load-bearing scaffolds. EtO sterilisation, while more polymer-friendly, requires aeration to remove residual gas and adds processing time and cost. Steam sterilisation (autoclaving) is generally contraindicated for PHAs due to hydrolysis and thermal degradation above the polymer’s melting temperature. Importantly, sterilisation effects are formulation-dependent: the presence of plasticisers, ceramic fillers (e.g., hydroxyapatite), or blending with other polymers can alter the polymer’s response to sterilisation. Therefore, veterinary device developers must validate sterilisation protocols for each specific PHA formulation and device geometry, rather than extrapolating from published data on neat polymers. This validation step is not merely a technical detail but a regulatory prerequisite for any implantable device, yet it remains conspicuously absent from most veterinary PHA literature.

### 5.7. Endotoxin Contamination and Purity Requirements for Biomedical-Grade PHA

A second important translational barrier that receives insufficient attention in the veterinary PHA literature is endotoxin contamination. Endotoxins are lipopolysaccharides (LPS) derived from the outer cell wall of Gram-negative bacteria, the very organisms most commonly used for industrial PHA production, including *Cupriavidus necator* (formerly *Ralstonia eutropha*), recombinant *Escherichia coli*, and Pseudomonas species. When introduced into the bloodstream or tissues, endotoxin triggers a potent inflammatory response, including fever, hypotension, and in severe cases, septic shock. For any implantable PHA device, endotoxin levels must be reduced to acceptable limits to avoid adverse local and systemic reactions.

For implantable medical devices such as sutures, meshes, and orthopaedic hardware, endotoxin limits are not expressed volumetrically (EU/mL) but rather as total endotoxin per device. In accordance with the regulations, devices that contact the cardiovascular or lymphatic systems must not exceed 20 EU/device; devices contacting cerebrospinal fluid must not exceed 2.15 EU/device. (The 0.5 EU/mL figure commonly cited in the literature applies to Water for Injection and parenteral fluids, not to solid implantable devices). For purified PHA polymer powders intended for device fabrication, limits are typically expressed as EU per gram of material, with thresholds such as <1 EU/g being a common target for biomedical-grade material [60,61].

Achieving these levels requires dedicated purification steps beyond simple polymer recovery. Early work by Lee and colleagues demonstrated that PHB recovered by chloroform extraction from Gram-negative bacteria contained less than 10 EU per gram of polymer, irrespective of bacterial strain or initial PHB content [57]. More aggressive purification, such as NaOH digestion of recombinant E. coli-produced PHB, reduced endotoxin levels to below 1 EU/g when digestion time and NaOH concentration were optimised.

For medium-chain-length PHAs, Wampfler et al. developed a non-chlorinated solvent extraction process using methyl tert-butyl ether (MTBE), ethyl acetate, or acetone, followed by activated charcoal filtration [61]. Filtration of the extract through activated charcoal yielded colourless polymers with less than one endotoxin unit per gram. More recently, affinity-based approaches have been explored, including the use of recombinant human lipopolysaccharide-binding protein fused to the PHA granule-associated protein PhaP, immobilised on PHB particles, achieving endotoxin removal efficiencies exceeding 90% [62].

These purification methods, however, come with significant economic and environmental costs. Most high-purity PHA downstream processes rely on organic solvents and chemicals, contributing substantially to overall production costs and environmental footprint [57]. The separation step itself, typically involving centrifugation, precipitation, evaporation, and filtration, adds further complexity [63]. For veterinary applications, where cost sensitivity is particularly acute in livestock medicine, there exists a fundamental tension between achieving biomedical-grade purity (with its associated expense) and producing a lower-cost “veterinary-grade” material that may tolerate higher endotoxin thresholds depending on the implantation site and duration of tissue contact. This distinction, between human-equivalent and veterinary-equivalent purity grades, has not been systematically addressed in the literature, yet it remains a crucial translational and regulatory question. For any veterinary PHA device intended for implantation, the endotoxin burden must be characterised, and purification protocols must be validated, as part of the preclinical safety dossier.

## 6. Clinical and Preclinical Applications in Veterinary Medicine

This section synthesises the evidence base by application area, distinguishing (a) studies whose primary purpose was veterinary clinical translation, (b) large-animal studies conducted as a surrogate for human clinical translation but germane to veterinary practice by virtue of species and outcome, and (c) more exploratory or in vitro work relevant to future veterinary development. Where the four source drafts synthesised for this review reported specific numerical outcomes (percentage closure rates, *p*-values, cost-per-animal figures) attributed to non-traceable or apparently templated citations, those figures have been omitted rather than propagated; findings below are described qualitatively and referenced only where a verifiable primary or review source underpins the claim.

### 6.1. Wound Healing and Skin Applications

Biodegradable PHA-based dressings, films, hydrogel-like composites, and electrospun nanofibrous mats, have been investigated for their capacity to support a moist wound environment, provide a bacterial barrier, and progressively resorb without requiring removal, a practical advantage in veterinary settings where dressing changes may require sedation [40,46]. Electrospun PHB and PHBV nanofibres, owing to their extracellular-matrix-like architecture, have shown enhanced in vitro fibroblast and keratinocyte biocompatibility, supporting their rationale as a wound-dressing substrate [47,64]. Incorporation of antimicrobial agents, silver nanoparticles, antimicrobial peptides, or natural antimicrobials, into PHA matrices is an actively explored strategy for infection control in both companion-animal surgical wounds and livestock applications such as post-procedural teat or udder care, motivated substantially by the desire to reduce reliance on systemic or topical antibiotics in food-producing animals [65]. Chitosan/PHB and collagen/PHA composite dressings improve hydrophilicity and cell adhesion relative to unmodified PHB films, addressing one of the principal limitations of native PHA surfaces in wound-contact applications [47].

*Species considerations*: Dogs and cats are the most frequent subjects of exploratory companion-animal wound studies given their clinical accessibility; large-animal (equine, bovine, ovine) wound-healing work more often occurs in the context of surgical incision management or teat/udder health rather than dedicated PHA-dressing trials, and dedicated, adequately powered veterinary clinical trials of PHA dressings remain scarce in the peer-reviewed literature.

### 6.2. Tissue Engineering and Regenerative Medicine

**Figure 4:**
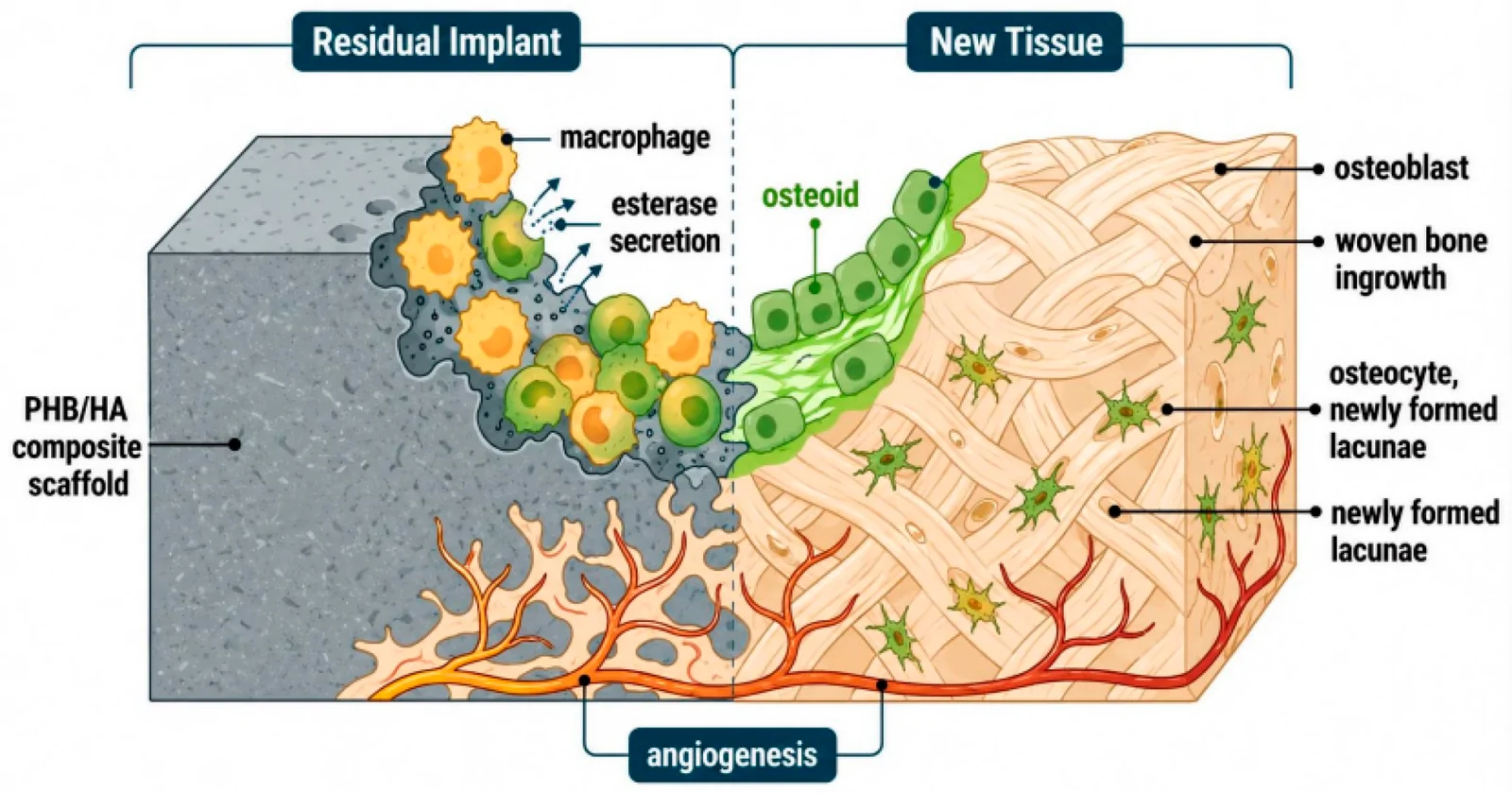
Representative histological schematic of PHA/HA composite scaffold integration in an osteochondral defect model, showing new bone ingrowth alongside residual, partially degraded polymer. The image was created by using FigureLabs (cloud-based platform, FigureLabs, available online: https://figurelabs.ai/).

**Bone and cartilage**. PHB and PHBV, alone or as HA composites, have long been investigated as resorbable, osteoconductive scaffold materials, dating to early work demonstrating in vivo bone-implant integration of PHB and PHB/HA composites in animal models [48,66]. Sheep are a commonly used large-animal model for orthopaedic and osteochondral defect studies, including for materials other than PHAs, primarily due to their size—which permits evaluation of implants under physiologically relevant mechanical loads—and their docility, which facilitates surgical manipulation and postoperative management. However, it is important to note that the ovine skeleton differs fundamentally from the human (and canine) skeleton in bone microarchitecture: sheep have a predominance of plexiform (primary) bone with fewer secondary osteons and lower remodelling activity compared to the haversian bone typical of humans and dogs. While older sheep do develop some haversian remodelling, the overall bone turnover rate and structural properties are not directly equivalent to those of humans. Therefore, the ovine model is best suited for mechanical and surgical feasibility studies rather than as a direct proxy for human or canine bone healing biology. This distinction is essential when interpreting preclinical data for translation to veterinary orthopaedics, particularly for companion animals such as dogs, whose bone physiology more closely resembles that of humans [46]. A representative histological schematic of PHA/HA composite scaffold integration in an osteochondral defect is shown in Figure 4.

**Cardiovascular: tissue engineering**. Among the most rigorously documented large-animal applications of PHA-based materials is their use as a scaffold component in tissue-engineered heart valves and vascular conduits, where P4HB-coated polyglycolic acid (PGA) scaffolds seeded with autologous vascular or valvular cells have been implanted in lamb models of the pulmonary circulation, demonstrating scaffold degradation with concurrent neotissue formation over the study period [67,68]. It is important to note that this body of work, while conducted in a veterinary species (sheep/lambs) and directly informative for veterinary cardiovascular tissue engineering, was designed primarily to de-risk a future paediatric human heart-valve therapy rather than as a veterinary clinical intervention; it nonetheless constitutes some of the most methodologically rigorous large-animal PHA data available and is directly transferable to veterinary cardiovascular device development.

**Peripheral: nerve and tendon**. PHA-based nerve guidance conduits and electrospun tendon/ligament scaffolds have been explored predominantly at the in vitro and small-animal (rodent, rabbit) level; translation to clinically relevant veterinary large-animal tendon or nerve injury models is comparatively immature and represents a clear research gap rather than an established application [9].

### 6.3. Drug-Delivery Systems

The sustained, tuneable degradation of PHAs makes them mechanistically attractive as carriers for controlled-release veterinary formulations, an area with particular relevance to livestock medicine, where reducing dosing frequency (and associated handling stress and labour cost) is a practical benefit. PHA microspheres and implants have been explored as vehicles for sustained release of antimicrobials, anti-inflammatory agents, and reproductive hormones, exploiting the same surface-erosion degradation mechanism that governs structural PHA implants [63,69,70]. This is a scientifically relevant and mechanistically well-founded application area, but—consistent with the broader gap identified in this review—peer-reviewed, adequately controlled veterinary clinical data specifically validating PHA-based sustained-release formulations (as opposed to formulations based on other, more established biodegradable carriers such as PLGA) remain insufficient, and claims of specific efficacy improvements over conventional formulations should be treated as hypothesis-generating pending confirmatory veterinary trials.

### 6.4. Surgical Devices: Sutures, Meshes, and Adhesion Barriers

This is the application area with the strongest existing translational precedent, via human-medicine devices whose material basis (P4HB) is directly relevant to veterinary use. P4HB-based monofilament sutures and soft-tissue reinforcement meshes (e.g., Phasix^TM^ Mesh, Davol Inc., Warwick, RI, USA, resorbing over 12–18 months; GalaFLEX^TM^ Scaffold, Tepha, Inc., Lexington, MA, USA, resorbing over 18–24 months) have achieved regulatory clearance and clinical use in human surgery, combining tensile strength retention profiles and resorption timelines comparable to or exceeding some conventional absorbable sutures, with reduced long-term foreign-body response reported in comparative studies [41,43]. The manufacturing and sterilisation groundwork for these materials is already substantially completed for human applications, making them the most credible near-term pathway for veterinary PHA device translation. Dedicated veterinary clinical trials of these commercial devices, however, are few; most veterinary use to date remains off-label extrapolation from human indications, and no P4HB device has yet received dedicated veterinary regulatory clearance (e.g., FDA-CVM or EMA-VMP approval). This off-label use is legally permissible under U.S. and European regulatory frameworks, as veterinarians may use human-cleared devices when no veterinary-specific alternative exists; however, it remains an extrapolation that carries inherent uncertainties, as discussed below.

Adhesion barriers and orthopaedic pins/screws fabricated from PHB or PHB composites have likewise been explored since the early 1990s, with in vivo animal studies demonstrating osseointegration and gradual resorption of PHB-based bone-fixation devices without acute adverse tissue response, providing early proof-of-concept for resorbable PHA orthopaedic hardware [48].

The translational pathway proposed above, leveraging human P4HB device regulatory data, requires an explicit methodological caveat. While the manufacturing, sterilisation, and regulatory-toxicology foundation established for human devices [41,43] offers a substantial starting point, it does not eliminate the need for species-specific validation. As will be discussed in Section 5, the local tissue response to PHA degradation products—particularly 4-hydroxybutyrate from P4HB—may differ across species with markedly different immune physiology, metabolic clearance pathways, and tissue turnover rates (e.g., variations in hepatic enzymatic capacity or systemic metabolic rates between ruminants and monogastrics) [54]. The human regulatory precedent should therefore be viewed as a foundation, not a substitute for veterinary-specific data. Any translation of P4HB-based devices to veterinary use must include: (i) species-specific in vivo degradation kinetics to confirm that resorption timelines match the target species’ healing rates; (ii) local tissue response profiling in the intended veterinary species (e.g., equine, canine, bovine) to rule out unexpected inflammatory or foreign-body responses; and (iii) metabolic clearance studies to ensure that degradation products (e.g., 4HB) do not accumulate to pharmacologically active levels in species with different metabolic rates. Without these data, off-label use of human-cleared P4HB devices remains an extrapolation that carries inherent immunological and metabolic uncertainty.

A separate but related caveat applies to all pre-clinical degradation data discussed in this review: the half-life values reported in rodent or ovine models are not directly transferable to companion animals or livestock without allometric correction. For example, the 12–18-month resorption time reported for Phasix^TM^ Mesh in human clinical use [41] does not guarantee an identical timeline in a canine or equine patient; the implant may persist longer or shorter depending on the recipient species’ metabolic rate and local tissue microenvironment. Until species-specific pharmacokinetic and degradation studies are conducted, device designers and clinicians should treat all published half-life ranges as order-of-magnitude estimates requiring validation in the target species.

### 6.5. Species-Specific Considerations

• *Dogs and cats*: The most accessible companion-animal species for exploratory wound-dressing and soft-tissue-device studies; body-size range necessitates scalable device dimensions, and owner-driven demand for reduced-intervention (single-surgery) procedures is an important clinical driver.
• *Horses*: Orthopaedic and tendon/ligament applications are of high interest given the frequency and economic impact of musculoskeletal injury in performance horses, but large equine bone/joint defects impose substantial mechanical demands that may exceed the load-bearing capacity of unreinforced PHA scaffolds, favouring HA- or fibre-reinforced composites.
• *Cattle and small ruminants*: Cost-per-animal constraints are the dominant translational barrier; applications must be inexpensive to be viable at herd scale, favouring simple formulations (e.g., antimicrobial-loaded films or sutures) over complex composite or 3D-printed devices, and mastitis-related applications are of particular interest given the antimicrobial-resistance context in dairy production.
• *Wildlife and exotic species*: Essentially unstudied with respect to PHA-specific biocompatibility; constitutes both a research gap and, given the diversity of physiology involved, a case where broad claims of “universal biocompatibility” across phylogenetically diverse taxa should be treated with particular caution.
• *Canine osteoarthritis*: An owner-reported outcomes study evaluated the safety and efficacy of intra-articular 2.5% polyacrylamide hydrogel (iPAAG) injection in dogs with osteoarthritis [71]. The study demonstrated that iPAAG was well-tolerated and associated with reduced osteoarthritis signs and decreased use of adjunctive therapies. However, iPAAG is a non-biodegradable mechanical cushion—it acts as a permanent “liquid prosthesis” providing viscosupplementation, not as a drug-delivery vehicle. Its success therefore does not provide direct mechanistic evidence for a PHA-based injectable system, which would function as a biodegradable pharmacological delivery platform. The appropriate comparator for PHA-based injectables is the established literature on biodegradable microspheres (e.g., PLGA- or PLA-based sustained-release formulations for intra-articular corticosteroids or disease-modifying drugs) [71]. While the iPAAG study provides useful evidence of procedural feasibility (e.g., that intra-articular injections are well tolerated in canine patients), it should not be interpreted as validating the pharmacological or degradation-based rationale for PHA injectables. The development of PHA-based injectable formulations for canine osteoarthritis would require dedicated preclinical studies assessing drug release kinetics, local tissue tolerance to 4-hydroxybutyrate degradation products, and efficacy against relevant osteoarthritis end points—building on the broader biomaterial’s literature for biodegradable drug delivery rather than on the polyacrylamide viscosupplementation model.

The evidence matrix of PHA applications across target veterinary species, categorised by evidence type, is shown in Table 4.

Differences in immune physiology, metabolic processing, and clearance mechanisms may potentially influence the degradation kinetics of PHA-based biomaterials. These processes differ among animal species and could, in principle, modify the local microenvironment surrounding the polymer and consequently influence degradation. These mechanisms should be distinguished from experimentally demonstrated species-specific effects. At present, direct comparative evidence establishing that differences in immune physiology, metabolism, or tissue turnover produce quantitatively different PHA degradation rates or degradation-product accumulation across animal species remains insufficiently investigated. Experimental findings obtained in animal models should therefore be interpreted within the context of that model and should not be directly extrapolated to other species, without considering differences in polymer characteristics, implantation site, tissue response, degradation kinetics, and systemic clearance.

## 7. Challenges, Limitations, and Future Directions

*Production cost and scalability*. Recent 2025–2026 analyses confirm that PHA production costs remain a substantial barrier to widespread commercialisation, despite extensive research in bioprocess development and the use of inexpensive waste streams [35,38]. A comprehensive review by Pan et al. [35] summarised current techno-economic estimates: PHA production costs are approximately $4–6 per kilogram, compared to $1–2 per kilogram for petrochemical plastics. This represents a 2- to 6-fold cost premium over commodity plastics, with roughly 50% of the cost attributable to the fermentation substrate [35]. PHA production costs thus remain substantially above those of PLA, let alone commodity plastics, although the gap has narrowed with process intensification and feedstock innovation. The minimum selling price for PHA is estimated at $4–8/kg, with product prices varying between €7 and 12/kg depending on production volumes [35]. The same review identified three principal cost-reduction strategies: (i) utilising waste resources as feedstocks, (ii) employing extremophiles as producer strains to enable non-sterile production, and (iii) applying genetic engineering to enhance PHA yields.

*Batch-to-batch and inter-laboratory variability*. Because PHA molecular weight, monomer ratio, crystallinity, and residual endotoxin/impurity content are all sensitive to fermentation and purification conditions, reported “PHA” materials across the literature are frequently not directly comparable, complicating both meta-analysis of existing data and regulatory standardisation [32,34,59,73]. Cho et al. [59] reviewed separation and purification technologies in PHA manufacturing, emphasising that rigorous quality control and standardised characterisation protocols are prerequisites for reliable biomedical translation. Establishment of standardised characterisation protocols (analogous to pharmacopoeial standards for other implantable biomaterials) is a prerequisite for reliable veterinary translation.

*Regulatory pathways*. According to a 2025 bibliometric and biotechnological review that synthesises primary regulatory records, formulations containing a high proportion of P4HB have received FDA approval for specific clinical applications, highlighting the translational significance of PHAs in regenerative medicine [74]. The same review noted that with advances in microbial biotechnology and regulatory support, PHAs are well positioned to become key materials in the development of sustainable biomedical devices. However, unlike human medical devices, veterinary biomaterials in most jurisdictions lack a dedicated, harmonised regulatory framework specifically addressing biodegradable implant materials, and veterinary approval often proceeds via extrapolation from human device data or country-specific veterinary drug/device authorities with variable evidentiary requirements [41,59]. This regulatory ambiguity is a substantial and underappreciated barrier to systematic veterinary PHA translation, distinct from the scientific/technical challenges above.

*Long-term* in vivo *safety data, particularly in large animals*. Most rigorous in vivo PHA degradation and biocompatibility data derive from short-duration rodent studies; large-animal (equine, bovine) long-term (>6–12 month) degradation and chronic-tissue-response data remain sparse, which is a significant gap given that clinically relevant implant lifetimes for orthopaedic applications in large animals often exceed a year [39,48,51]. A study by Puppi et al. [74] described the fabrication and characterisation of poly[(R)-3-hydroxybutyrate-co-(R)-3-hydroxyhexanoate] (PHBHHx) tissue engineering scaffolds with anatomical shape and customised porous structure using computer-aided wet-spinning, demonstrating the feasibility of producing patient-specific (or animal-specific) PHA scaffolds with precisely controlled pore architecture.

*Species-specific immunological and metabolic variability*. The degradation product profile and local tissue response to PHA implants may plausibly differ across species with markedly different immune and metabolic physiology (e.g., ruminant forestomach microbiota versus monogastric gut, or avian versus mammalian immune architecture), yet comparative immunological studies across veterinary species are essentially absent from the literature [46,75], representing both a scientific gap and a caution against over-generalising biocompatibility claims established in one species to another.

**Figure 5:**
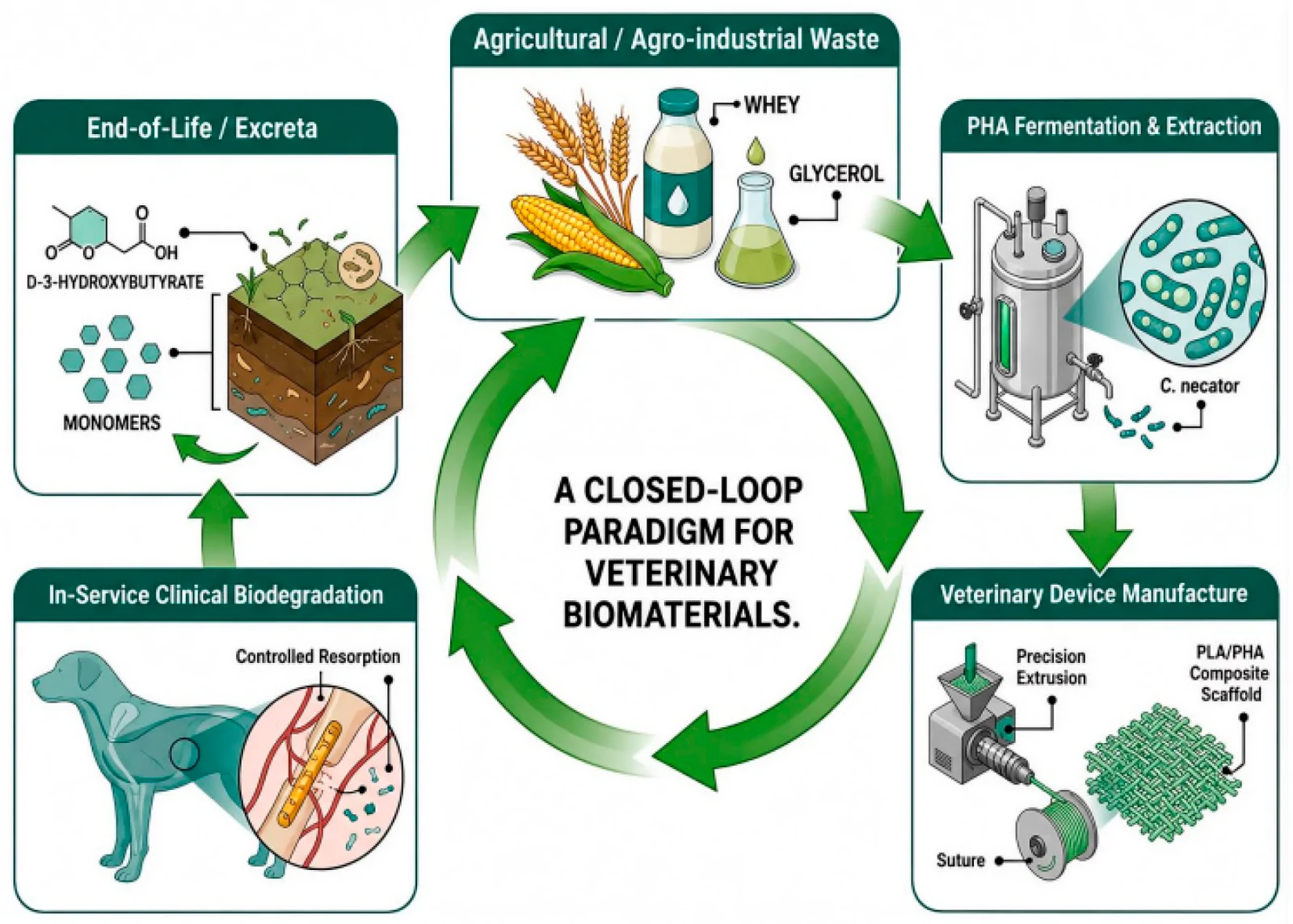
Conceptual circular-economy flow diagram linking agricultural/agro-industrial waste feedstocks → PHA fermentation → veterinary device manufacture → in-service biodegradation → soil/compost reintegration. The image was created by using FigureLabs (cloud-based platform, FigureLabs, available online: https://figurelabs.ai/).

*Future directions*. Priority research areas include: (i) standardised, veterinary-specific biocompatibility and degradation testing protocols across major target species; (ii) cost-competitive production from regionally available agricultural waste streams adapted to veterinary-scale (rather than pharmaceutical-scale) purity requirements, potentially permitting a lower-cost “veterinary grade” alongside a higher-cost, more rigorously purified “human-equivalent grade” [35,37,59]; (iii) systematic exploitation of the existing regulatory and manufacturing precedent for P4HB-based human surgical devices as a fast-track pathway to veterinary-labelled equivalents [41,74]; (iv) rigorous, adequately powered veterinary clinical trials (rather than case series or small pilot cohorts) for the most mechanistically promising applications identified above (antimicrobial wound dressings, resorbable sutures/meshes, orthopaedic composites) [73,74]; and (v) life-cycle and circular-economy analyses that quantify, rather than assert, the environmental benefit of PHA substitution in veterinary/agricultural settings. A conceptual circular-economy flow diagram for PHA-based veterinary devices is presented in Figure 5. Future efforts should prioritise efficient pretreatment, metabolic engineering of resilient strains, intelligent bioprocess design, and comprehensive sustainability assessments to integrate PHA production into a circular bioeconomy [35,36,37]. Beyond implantable devices, PHA-based nutritional supplementation is emerging in aquaculture. A 2025 study in common carp demonstrated that dietary PHB at 1.00% inclusion improved weight gain and feed conversion, though higher inclusion (5.00%) showed less promising metabolic markers [76]. This application—using PHB as a feed additive rather than as a structural biomaterial—highlights the versatility of PHAs but is distinct from the medical-device focus of this review.

Key translational barriers for veterinary PHA devices, alongside proposed mitigation strategies and responsible stakeholder groups, are outlined in Table 5.

## 8. Conclusions

Polyhydroxyalkanoates possess a beneficial combination of biodegradability, with adjustable mechanical and degradation properties, and a growing, if still human-medicine-centred, regulatory and manufacturing precedent, chiefly through P4HB-based resorbable sutures and meshes. Veterinary medicine presents a scientifically and economically compelling application space for these materials, particularly for resorbable sutures and soft-tissue devices, antimicrobial wound dressings, and composite orthopaedic scaffolds, with the strongest existing evidence base concentrated in cardiovascular tissue-engineering work conducted in ovine models (albeit largely as a human-translational surrogate) and in the general orthopaedic and wound-healing proof-of-concept literature. However, dedicated veterinary-clinical validation, as distinct from extrapolation from rodent or human-translational large-animal data, remains the field’s principal shortfall. Closing this gap will require standardised characterisation and testing protocols, cost-effective and consistent biomanufacturing routes suited to veterinary-scale economics, clearer veterinary-specific regulatory pathways, and rigorously designed clinical trials in the target species themselves. Achieving these will determine whether PHAs move from a scientifically robust biomaterial platform to a clinically established component of routine veterinary practice.

## Figures and Tables

**Table 1:** Comparison of biodegradable polymers currently used or under investigation in veterinary medicine.

Polymer	Key Properties	Veterinary Applications	Advantages	Limitations	References
**PDS**	• Monofilament • Retains strength 6–8 weeks • Complete absorption in approx. 180 days	• Soft tissue approximation • Vascular ligation • General surgery	• Predictable degradation • Excellent handling • Reduced bacterial adherence	• Petrochemical-derived • Not renewable • Degradation products not natural metabolites	[4,5]
**PGA**	• Braided multifilament • Absorption 60–90 days • Bulk hydrolysis	• Tissues with rapid healing • Drug-delivery scaffolds	• Well-characterised • Regulatory acceptance • Adapted degradation with PLGA	• Acidic degradation products • Bulk erosion • Local inflammation risk	[6]
**PLGA**	• Copolymer • Adjustable degradation • Bulk hydrolysis	• Drug-delivery systems • Tissue engineering scaffolds	• Widely studied • FDA-cleared for multiple implantable devices (e.g., surgical meshes, bone fixation pins, resorbable scaffolds) [19] • Versatile formulations	• Acidic degradation products • Batch-to-batch variability • Not naturally occurring metabolites	[4,9,12]
**PLA**	• Semicrystalline • Absorption: months to years • Bulk hydrolysis	• Orthopaedic implants (TTA) • Resorbable fixation devices	• Renewable resource • Osteoconductive • Established infrastructure	• Degrades slowly (up to 4 years) • Less flexible than P4HB • Degradation products not natural metabolites	[7,8,10]
**PCL**	• Very slow degradation • Years for complete resorption	• Long-term scaffolds • Drug-delivery systems	• Stable over long periods • Well-characterised	• Too slow for many applications • Limited mechanical strength	[11]
**PHAs**	• Surface erosion • Degrades to natural metabolites • Wide property range (stiff to elastomeric)	• Sutures, meshes, wound dressings, orthopaedics, drug delivery	• Renewable feedstock • Non-toxic degradation products • Adjustable mechanics • Biocompatible	• Higher production cost • Limited veterinary regulatory precedent • Batch-to-batch variability • Endotoxin purification challenges	[1,8,9,13,14]

**Table 2:** Comparative physicochemical and mechanical properties of major PHA variants.

PHA Variant	Crystallinity (%)	Melting Temp (Tm, °C)	Glass Transition (Tg, °C)	Tensile Strength (MPa)	Elongation at Break (%)	Tensile Modulus (GPa)	Typical In Vivo Half-Life
**Bulk/Unoriented Material**							
**PHB**	55–70 ^1^	170–180	0–5	35–40	3–8	3.0–3.5	Very slow (>12–24 months)
**PHBV (10–20% HV)**	35–50	130–150	−10 to −5	20–30	15–50	1.5–2.5	Slow (9–18 months)
**P4HB**	30–45	55–61 ^2^	−50 to −45	30–40	100–300	0.05–0.1	Slow (12–24 months, formulation- and site-dependent)
**PHBHHx (10–12% HHx)**	20–35	110–125	−10 to −1	15–25	300–500	0.2–0.5	Moderate (6–12 months)
**Processed/Oriented Forms (e.g., sutures, meshes)**							
**P4HB (fibre-drawn)**	- ^3^	-	-	500–800+	15–30	-	Slow (12–24 months; formulation- and site-dependent)

Footnotes: ^1^ PHB crystallinity varies with processing and thermal history; 55–70% is typical for melt-processed samples [35]. ^2^ P4HB melting temperature varies with molecular weight and thermal history; representative range from [5,36]. ^3^ Crystallinity of fibre-drawn P4HB may differ from bulk values and is not reported here.

**Table 3:** Summary of PHA-modification strategies for veterinary applications.

Modification Strategy	Primary Mechanism	Representative Additive/Technique	Targeted Property Change	Principal Material Limitation
**Physical blending**	Macromolecular mixing via solution or melt processing	Chitosan, gelatine, PEG, PLA, PLGA	Reduces stiffness/brittleness; tunes degradation rate; improves hydrophilicity.	Thermodynamic phase separation; risk of interfacial mechanical failure.
**Chemical grafting**	Covalent attachment of functional chemical groups	Acrylic acid grafting; PEGylation; EDC/NHS coupling	Introduces specific reactive handles for bioactive ligand immobilisation.	Complex purification steps; potential toxicity from unreacted monomers/catalysts.
**Surface modification**	Superficial chemical or physical alteration without bulk change	Plasma treatment (O_2_, Ar); UV/ozone exposure	Drastically improves surface wettability (hydrophilicity) and initial cell attachment.	Modification layer is often transient; surface properties change over time (surface aging).
**Composite formation**	Integration of a distinct, functional secondary phase	Hydroxyapatite (HA); β-tricalcium phosphate (β-TCP); Bioglass	Matches bone mechanics (modulus); imparts osteoconductivity for rigid implants.	Higher melt viscosity complicates 3D printing; processing can alter polymer molecular weight.

**Table 4:** Summary of PHA applications across veterinary species with evidence classification.

Application Area	Companion Animals (Dogs, Cats)	Equine (Horses)	Ruminants (Cattle, Sheep, Goats)	References
**Wound healing and skin care**	• In vitro validation of canine fibroblast attachment on electrospun PHBV • Small pilot cohorts for superficial wound dressings	• Highly limited; mostly extrapolated from rodent models • High risk of dressing displacement in active patients	• Topical antimicrobial-loaded PHA films for bovine teat/udder health • Intramammary/teat-based antimicrobial delivery for mastitis prevention and treatment • Driven by antibiotic-reduction mandates	[47,52,64,65]
**Orthopaedic and cartilage repair**	• Finite element models and rodent defect proxies • Bone-fixation pins tested in small animal models	• High interest but sparse clinical data • Exceptional mechanical loads require high-fraction HA composites	• Extensive data using ovine (sheep) models for segmental bone defects • Note: Conducted primarily as human-translational proxies	[66,72]
**Cardiovascular tissue engineering**	• In vitro and small rodent vascular patch assays only	Virtually unstudied due to low clinical demand	• Strongest large-animal dataset • P4HB-coated PGA scaffolds for heart valves/conduits validated in growing lamb models	[67,68]
**Drug-delivery systems**	• Subcutaneous micro/nanoparticles for sustained analgesic or NSAID release (experimental) • Intra-articular polyacrylamide hydrogel injection well tolerated and reduces OA signs in dogs; demonstrates procedural feasibility of intra-articular delivery in canine patients	Exploratory applications for localised intra-articular drug delivery in joint lameness	Subcutaneous or intraruminal biodegradable implants for synchronised hormone/antimicrobial release	[11,28,71]
**Surgical devices (sutures, meshes)**	Off-label clinical adoption of human-cleared P4HB monofilament sutures/meshes in complex hernia repairs	Limited exploratory use in abdominal wall reinforcement (colic surgery laparotomy closures)	Evaluation of resorbable PHA sutures in production-animal soft tissue surgeries (e.g., caesarean sections)	[5,6,14,41,43]

**Table 5:** Key translational barriers for veterinary PHA devices and proposed mitigation strategies.

Challenge	Description	Proposed Mitigation Strategy	Responsible Stakeholders	References
**Production cost**	PHA costs $4–6/kg vs. $1–2/kg for conventional plastics; 50% cost from fermentation substrate	• Waste-feedstock valorisation (glycerol, whey, lignocellulose) • Mixed microbial consortia • Process intensification	• PHA manufacturers • Industrial bioprocess engineers • Agricultural waste management cooperatives	[24,29,34]
**Batch-to-batch variability**	Molecular weight, crystallinity, and monomer ratio vary with fermentation conditions	• Standardised characterisation protocols • Quality-by-design (QbD) approaches • Real-time process monitoring	• PHA quality control laboratories • Regulatory bodies (USP, EP) • Analytical chemists	[25,32,70]
**Regulatory pathways**	No dedicated veterinary biodegradable implant framework; off-label use of human devices	• Develop veterinary-specific guidance • Leverage human device data (510(k) pathway) • Engage regulators early	• FDA-CVM, EMA-VMP • Veterinary device manufacturers • Veterinary professional associations (AVMA, FVE)	[41,59]
**Long-Term Safety Data**	Most in vivo data from rodent studies (<12 months); large-animal data sparse	• Equine/bovine long-term (>12 month) degradation studies • Multi-species biocompatibility testing	• Academic veterinary research centres • Veterinary clinical trial networks	[45,57,66]
**Species-Specific Variability**	Immunological and metabolic responses differ across species (e.g., ruminant vs. monogastric)	• Comparative immunology studies • Species-specific in vivo testing • Careful extrapolation from human data	• Comparative biologists • Veterinary pathologists	[77]
**Sterilisation**	Gamma irradiation degrades PHA; EtO adds cost and time; steam contraindicated	• Validate EtO for each formulation • Explore e-beam as alternative • Develop sterilisation-resistant formulations	• Medical device sterilisation service providers • PHA device manufacturers	[57]
**Endotoxin** **/Purity**	Gram-negative bacterial production introduces LPS; removal adds cost and complexity	• Optimise purification (NaOH digestion, activated carbon, affinity) • Consider “veterinary grade” purity tier	• Downstream processing engineers • Regulatory toxicologists	[59,61,62,63]

## Data Availability

No new data were created or analysed in this study. Data sharing is not applicable to this article.
